# Critical attributes of human early mesenchymal stromal cell-laden microcarrier constructs for improved chondrogenic differentiation

**DOI:** 10.1186/s13287-017-0538-x

**Published:** 2017-05-08

**Authors:** Youshan Melissa Lin, Jialing Lee, Jessica Fang Yan Lim, Mahesh Choolani, Jerry Kok Yen Chan, Shaul Reuveny, Steve Kah Weng Oh

**Affiliations:** 1 Bioprocessing Technology Institute, Agency for Science, Technology and Research (A*STAR), 20 Biopolis Way, #06-01 Centros, Singapore, 138668 Singapore; 2 Experimental Fetal Medicine Group, Department of Obstetrics and Gynaecology, Yong Loo Lin School of Medicine, National University Health System, 1E Kent Ridge Road, NUHS Tower Block Level 12, Singapore, 119228 Singapore; 3 Department of Reproductive Medicine, KK Women’s and Children’s Hospital, 100 Bukit Timah Road, Singapore, 229899 Singapore; 4 Cancer and Stem Cell Biology Program, Duke-NUS Graduate Medical School, 8 College Road, Singapore, 169857 Singapore

**Keywords:** Cartilage, Cell therapy, Chondrogenic differentiation, Mesenchymal stromal cells, Microcarrier

## Abstract

**Background:**

Microcarrier cultures which are useful for producing large cell numbers can act as scaffolds to create stem cell-laden microcarrier constructs for cartilage tissue engineering. However, the critical attributes required to achieve efficient chondrogenic differentiation for such constructs are unknown. Therefore, this study aims to elucidate these parameters and determine whether cell attachment to microcarriers throughout differentiation improves chondrogenic outcomes across multiple microcarrier types.

**Methods:**

A screen was performed to evaluate whether 1) cell confluency, 2) cell numbers, 3) cell density, 4) centrifugation, or 5) agitation are crucial in driving effective chondrogenic differentiation of human early mesenchymal stromal cell (heMSC)-laden Cytodex 1 microcarrier (heMSC-Cytodex 1) constructs.

**Results:**

Firstly, we found that seeding 10 × 10^3^ cells at 70% cell confluency with 300 microcarriers per construct resulted in substantial increase in cell growth (76.8-fold increase in DNA) and chondrogenic protein generation (78.3- and 686-fold increase in GAG and Collagen II, respectively). Reducing cell density by adding empty microcarriers at seeding and indirectly compacting constructs by applying centrifugation at seeding or agitation throughout differentiation caused reduced cell growth and chondrogenic differentiation. Secondly, we showed that cell attachment to microcarriers throughout differentiation improves cell growth and chondrogenic outcomes since critically defined heMSC-Cytodex 1 constructs developed larger diameters (2.6-fold), and produced more DNA (13.8-fold), GAG (11.0-fold), and Collagen II (6.6-fold) than their equivalent cell-only counterparts. Thirdly, heMSC-Cytodex 1/3 constructs generated with cell-laden microcarriers from 1-day attachment in shake flask cultures were more efficient than those from 5-day expansion in spinner cultures in promoting cell growth and chondrogenic output per construct and per cell. Lastly, we demonstrate that these critically defined parameters can be applied across multiple microcarrier types, such as Cytodex 3, SphereCol and Cultispher-S, achieving similar trends in enhancing cell growth and chondrogenic differentiation.

**Conclusions:**

This is the first study that has identified a set of critical attributes that enables efficient chondrogenic differentiation of heMSC-microcarrier constructs across multiple microcarrier types. It is also the first to demonstrate that cell attachment to microcarriers throughout differentiation improves cell growth and chondrogenic outcomes across different microcarrier types, including biodegradable gelatin-based microcarriers, making heMSC-microcarrier constructs applicable for use in allogeneic cartilage cell therapy.

**Electronic supplementary material:**

The online version of this article (doi:10.1186/s13287-017-0538-x) contains supplementary material, which is available to authorized users.

## Background

Articular cartilage is a connective tissue that surrounds synovial joints [1–5]. It comprises chondrocytes and their secreted, specialized extracellular matrix that is rich in Collagen II fibers and glycosaminoglycans (GAGs) [1–5]. Its main biological functions are to allow weight-bearing and mechanical stress without being distorted, reduce friction to facilitate movement, and absorb shock [1–5]. However, it is highly susceptible to injury and degeneration as it does not regenerate well, leading to many disorders such as osteoarthritis which is one of the more prominent causes of immobility and pain globally [4, 6–8]. Existing therapeutic options to treat cartilage defects and deterioration are limited, particularly for allografts due to the inadequate supply of cadaver tissue [4, 6–8]. Therefore, researchers are exploring whether tissue engineering methods can be employed to create articular cartilage-like tissue in vitro that can be used for potential allogeneic cartilage cell therapy and tissue replacement [8–13].

Due to the regenerative abilities and multipotent nature of stem cells that allow them to differentiate into various cell and tissue types, there is huge interest to drive stem cells to differentiate along the chondrogenic lineage into chondrocyte-like cells in order to create articular cartilage-like tissue in vitro for eventual cartilage tissue replacement [9–13]. Among the many different types of stem cells, human mesenchymal stromal cells (hMSC) are an attractive stem cell population for cartilage cell therapy due to their relative ease of isolation, safety, and ethical acceptance [14–16]. In particular, human early mesenchymal stromal cells (heMSC) are good candidates for allogeneic cell therapy because they are more plastic, have better growth rates, and are slower to senesce than adult hMSC [17–19].

Recent studies have estimated that at least 1.5 to 4.5 × 10^7^ cells per patient are required for cartilage regeneration, meaning minimum lot sizes necessary for stem cell expansion need to be at least 100 × 10^9^ cells [20–24]. To enable such large-scale production of stem cells, it is practical to employ microcarrier-based technologies for cell expansion instead of using conventional static tissue culture plastic such as cell stacks [20]. Microcarrier-based cell expansion platforms are already used commercially in industrial-scale bioreactors to propagate mammalian cells such as Vero cells to produce vaccines [20, 25]. It involves cultivating adherent cells of interest on the surface of spherical particles—microcarriers—that can be suspended in culture medium by constant impeller agitation to create a homogenous cell suspension culture system [20, 26]. They are advantageous over conventional static tissue culture plastic as they provide a higher surface to volume ratio which is more cost-effective in terms of yield per unit medium, occupy less physical space per unit yield, and are easier to scale-up production in bioreactors [20, 27]. Hence, many are now turning to microcarrier-based culture systems for stem-cell expansion, particularly to expand hMSC for allogeneic cartilage cell therapy.

Our group has recently demonstrated that heMSC expansion on microcarriers in agitated spinner cultures is not only useful for attaining large amounts of cells, but also beneficial in enhancing the osteogenic and chondrogenic potential of the cells when compared to cells harvested from conventional tissue culture plastic [28, 29]. In addition, we have also shown that biodegradable heMSC-PCL microcarrier constructs can be generated and transplanted in vivo for bone regeneration [30]. This advantage of utilizing microcarriers to improve the differentiation potential of MSC is further supported by work from other groups [31–33]. Applying and expanding upon these findings for cartilage cell therapy, our current study aims to explore: 1) whether continuous cell attachment to microcarriers throughout the course of chondrogenic differentiation would affect or even improve differentiation outcomes and, more importantly, 2) whether microcarriers can be used additionally as scaffold material to create biodegradable and biocompatible hMSC-microcarrier constructs that can be differentiated efficiently along the chondrogenic lineage in vitro for potential transplantation into the patient. This proposition is highly attractive because it streamlines the production bioprocess from cell expansion all the way to cell transplantation, removing the need for additional manipulations mid-process such as enzymatic treatment to harvest the cells off the microcarriers before implantation, which can affect cell growth and chondrogenic differentiation efficiency. To the best of our knowledge, the only publication that has described a similar attempt to differentiate hMSC-laden microcarrier constructs along the chondrogenic lineage in vitro has met with limited success [34].

To address these aims, a screen was performed to define the critical attributes needed to enable efficient chondrogenic differentiation of heMSC-Cytodex 1 constructs in vitro. We identified cell confluency, cell seeding number, and number of microcarriers per construct to be critical parameters. Critically defined heMSC-Cytodex 1 constructs generated with 10 × 10^3^ cells at 70% cell confluency with 300 microcarriers per construct induced the greatest fold-increase in cell growth and chondrogenic output. Most importantly, we demonstrated that these critical attributes can be applied across multiple microcarrier types, including biodegradable gelatin- and collagen-based microcarriers, to generate efficient heMSC-microcarrier constructs that displayed improved cell growth and chondrogenic output per construct and per cell, when compared to equivalent cell-only counterparts. Overall, these findings support the adoption of microcarrier-based technologies not only for large-scale hMSC expansion, but also for advancing tissue engineering efforts to create efficient hMSC-microcarrier chondrogenic constructs for allogeneic cartilage cell therapy and tissue replacement.

## Methods

### heMSC expansion on static tissue culture plastic

heMSC were isolated, characterized, and approved for use by the Domain Specific Review Board of the National Healthcare Group in Singapore (DSRB-2006-00154), as previously described [35]. heMSC (passage 8 to 9) were plated at a density of 2400 to 2800 cells/cm^2^ in either noncoated or gelatin-coated T175 cm^2^ cell culture flasks or Nunc™ EasyFill™ Cell Factory™ Systems in MSC growth medium consisting of minimum essential medium α, 10% vol/vol fetal bovine serum (FBS) and 1% vol/vol penicillin-streptomycin (all from Gibco). Gelatin (0.1%; STEMCELL Technologies) was used to coat the surfaces of tissue culture plastic for 2 h at room temperature before cells were plated. heMSC were propagated at 37 °C in a 5% CO_2_ humidified incubator (Thermo Scientific) for 3 to 4 days to achieve about 70% confluency with a medium change every 2 to 3 days. Cells were harvested with 0.25% Trypsin-EDTA (Gibco) for 5 mins at 37 °C. Cell viability and count assays were performed using the NucleoCounter® NC-3000 (Chemometec), according to the manufacturer’s instructions.

### Preparation of microcarriers

Cytodex 1 (GE Healthcare), Cytodex 3 (GE Healthcare), SphereCol (Advanced BioMatrix), and Cultispher-S (Sigma) microcarriers were prepared in accordance with the manufacturer’s instructions. Cytodex 1, Cytodex 3, and Cultispher-S were sterilized by autoclaving at 121 °C for 20 min while SphereCol was supplied in sterile form. All microcarriers were washed three times in MSC growth medium before use. Characteristics of the distinct microcarriers used are summarized in Table 1.

**Table 1:** Characteristics of microcarriers tested and their respective seeding conditions per heMSC-microcarrier construct at day 0 of differentiation

	Cytodex 1	Cytodex 3	SphereCol	Cultispher-S
Microcarrier characteristics				
Diameter (μm)	147–248	141–211	100–400	130–380
Matrix	Dextran	Dextran	Type I Collagen	Gelatin
Charges/coating	Positively charged	Denatured collagen	–	–
Porosity	Microporous	Microporous	Microporous	Macroporous
Biodegradability	No	No	Yes	Yes
Surface area (cm^2^/mg dry weight)	4.40	2.70	–	15.0
heMSC-microcarrier construct-seeding conditions (spinner culture, data per construct)				
Cell confluency	70%	70%	–	–
Total cell number	10.1 × 10^3^	10.1 × 10^3^	–	–
Microcarrier number	300	300	–	–
heMSC-microcarrier construct-seeding conditions (shake flask culture, data per construct)				
Cell confluency	70%	70%	70%	70%
Total cell number	10.1 × 10^3^	8.88 × 10^3^	8.88 × 10^3^	30.8 × 10^3^
Microcarrier number	300	300	300	50

heMSC human early mesenchymal stromal cell

### Initial generation of heMSC-microcarrier constructs from cells expanded in spinner cultures or cells attached by the 1-day attachment process in shake flask culture

For spinner cultures, 4.8 × 10^4^ heMSC were seeded onto either 2.7 mg/ml Cytodex 1, or 4 mg/ml Cytodex 3 microcarriers, which is equivalent to 4 cells/bead ratio. Cells were cultivated in 500 ml disposable spinner flasks (Corning) at an agitation rate of 30 to 40 rpm using MSC growth medium for 7 days. A 50% medium change was done every other day. Cell-covered microcarriers from spinner culture at either day 3 (43% confluency), day 5 (68% cell confluency), or day 7 of the growth phase (95% cell confluency) were used to generate heMSC-microcarrier constructs for chondrogenic differentiation, as described below.

For shake flask cultures to achieve 70% cell confluency on each bead of the four types of microcarriers, 10.1, 8.88, 8.88, and 61.7 × 10^5^ heMSC harvested from tissue culture plastic were seeded into 25.0 ml MSC growth medium per microcarrier type per flask containing 6.98 mg Cytodex 1, 10.0 mg Cytodex 3, 0.273 ml SphereCol stock solution (1.10 × 10^5^ microcarriers/ml), and 12.5 mg Cultispher-S microcarriers, respectively. The amount of cells seeded per flask was calculated as: microcarrier amount in mg × microcarrier surface area in cm^2^/mg (Table 1) × 0.7 (70% cell confluency) × 4.7 × 10^4^ cells/cm^2^ (100% cell confluency). Cells were cultivated in 125 ml disposable Erlenmeyer flasks (Corning) at an agitation rate of 75 rpm with MSC growth medium for 1 day before seeding into heMSC-microcarrier constructs.

### Chondrogenic differentiation

All heMSC-microcarrier constructs derived from either spinner or shake flask cultures and cell-only pellets derived from tissue culture plastic (at 70% confluency) were generated by seeding cells attached to microcarriers or cells only in clear round-bottom ultra-low attachment 96-well plates (Corning) at 1 construct or 1 pellet per well in MSC growth medium at day 0 of differentiation. After 1 day, they were then differentiated in chondrogenic differentiation medium containing DMEM-high glucose (Gibco), 1 mM sodium pyruvate (Gibco), 100 nM dexamethasone (Sigma), 0.1 mM l-ascorbic acid-2-phosphate (Sigma), 1% vol/vol ITS + 1 (Sigma), l-proline (Sigma), 1% vol/vol penicillin/streptomycin (Gibco), and 100 ng/ml recombinant human BMP2 (CHO-derived; R&D Systems), with a medium change every 2 to 3 days for a total of either 21 days for the screening study or 28 days for all other experiments.

The differentiating culture was tested at day 21 for the screening study and weekly for 28 days for all other experiments.

### DNA, GAG, and Collagen II content evaluation

All heMSC-microcarrier constructs and cell-only pellets were rinsed once with phosphate-buffered saline (PBS) before immediate storage at –80 °C. After thawing, the heMSC-microcarrier constructs and cell-only pellets were either digested with 0.125 mg/ml papain at 65 °C overnight for DNA and GAG quantification, or with 0.1 mg/ml pepsin at 4 °C over 2 nights followed by 0.1 mg/ml elastase digestion at 4 °C overnight for Collagen II evaluation. DNA quantification was performed using Quant-iT™ Picogreen® dsDNA Assay (Life Technologies), GAG measurement was performed using Blyscan Sulfated Glycosaminoglycan Assay (Biocolor), and Collagen II quantification was performed by ELISA against Type II Collagen (Chondrex), all in accordance with the manufacturer’s instructions. All fluorometric and optical readings were taken with a Tecan Infinite M200.

### Construct/pellet diameter evaluation

Brightfield images of hematoxylin and eosin (H&E)-stained constructs (generated via spinner culture) and relevant cell-only pellets were taken with an Eclipse Ni-E microscope (Nikon). Brightfield images of heMSC-microcarrier constructs (generated via shake flask culture) and relevant cell-only pellets were taken with either an Eclipse Ni-Ti microscope (Nikon) or EVOS® FL Imaging System (Life Technologies). Images were processed with NIS-Elements (Nikon) and ImageJ software was used to determine the diameter of constructs or pellets.

### Histological and immunocytochemical staining

heMSC-microcarrier constructs were rinsed once with PBS before fixing with 4% paraformaldehyde at 4 °C overnight. Fixed samples were cleared with histoclear and embedded in paraffin wax. Sections (5-μm thick) were cut in a slide series of 20 and stained either with H&E using Leica AutoStainer XL Automatic Slide Stainer, or manually with 0.1% Safranin O, Alcian Blue at pH 1.0. Immunocytochemistry with mouse Collagen Type II monoclonal antibodies (clone 6B3; Millipore) at 1:1000 dilution was performed using the Leica Bond™ Autostainer. Sections were then examined by light microscopy with an Eclipse Ni-E microscope (Nikon).

### Gene expression evaluation

Cell-only pellets (at least 20 per condition per time point) were manually homogenized in Trizol solution (Life Technologies) using OMNI TH Tissue Homogenizer with Omni Hard Tissue Tips (OMNI International). Microcarrier beads were removed with a 40-μm cell strainer (Greiner bio-one) before RNA extraction. RNA was extracted with the Direct-zol™ RNA Purification Kit in accordance with the manufacturer’s protocol (Zymo Research) and its concentration was measured by NanoDrop (Biofrontier Technology). cDNA was synthesized from 100 ng RNA per sample by the Maxima® First-Strand cDNA Synthesis Kit, as per the manufacturer’s protocol (Thermo Scientific Fermentas). Relative mRNA expression of chondrogenic marker genes were measured by quantitative real-time polymerase chain reaction (qRT-PCR) with TaqMan® Probe-Based Gene Expression Analysis (Life Technologies) on the 7500 Fast Real-Time PCR System (Applied Biosystems). The TaqMan® probes used in this study are listed in Additional file 1: Table S1. Comparative C_t_ values were analyzed with StepOne 7500 Software (Applied Biosystems). Relative mRNA expressions of target genes were calculated based on the 2^∆∆Ct^ formula after normalization to GAPDH values with reference to cell-only pellets at day 0 of differentiation.

### Statistical analyses

Data are expressed as mean ± standard deviations and were analyzed with the statistical software Prism 6 (GraphPad). Multiple comparisons among different conditions were compared statistically using ordinary one-way analysis of variance (ANOVA) with Tukey’s multiple comparisons test. Pairwise comparisons were compared statistically using a Student’s *t* test. For all statistical tests, *p* values less than 0.05 were considered significant.

## Results

Conventional methods for chondrogenic differentiation of heMSC are by expanding the cells as static monolayer cultures on tissue culture plastic followed by enzymatic dissociation and generation of suspended cell pellets, which are further differentiated along the chondrogenic lineage using chondrogenic medium supplemented with inducers such as TGFβ1/3 or BMP2 [18, 36–39]. We have shown previously that heMSC harvested from agitated microcarrier-spinner cultures displayed improved chondrogenic differentiation when compared to those generated from conventional static monolayer cultures on tissue culture plastic [29]. Expanding on this work, in this study we aim to test whether heMSC-microcarrier constructs containing heMSC-covered microcarriers can be generated to effectively undergo chondrogenic differentiation.

### Defining critical attributes that enable effective chondrogenic differentiation of heMSC-microcarrier constructs

**Fig. 1:**
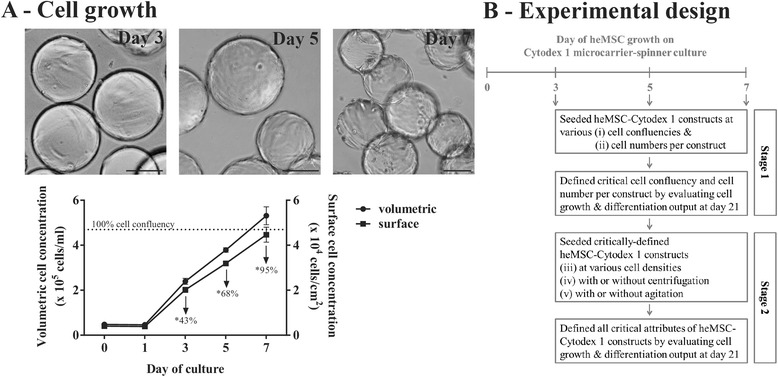
Evaluation of critical parameters required to achieve efficient chondrogenic differentiation of heMSC-Cytodex 1 microcarrier constructs. **a** Brightfield images (*scale bar* = 100 μm) and kinetics of heMSC growth on Cytodex 1 microcarriers in agitated spinner culture. Numbers indicate the cell confluency (*dotted line* represents 100% cell confluency of 4.7 × 10^4^ cells/cm^2^ as calculated from monolayer cultures). *Cell-laden microcarriers taken from spinner culture at the indicated time point were used to seed heMSC-Cytodex 1 constructs. **b** Schematic of experimental design. Stage 1: heMSC attached to Cytodex 1 microcarriers were seeded as chondrogenic heMSC-microcarrier constructs at either day 3 (early-log phase with 43% cell confluency), day 5 (mid-log phase with 68% cell confluency), or day 7 (late-log phase with 95% cell confluency), using different cell numbers per construct. Stage 2: heMSC-microcarrier constructs generated under critically defined conditions as identified at Stage 1 were evaluated for the effect of cell density (addition of empty microcarriers at seeding) or the effect of compaction (centrifugation at seeding or agitation throughout differentiation)

A screen to evaluate five potential factors that can affect the chondrogenic differentiation efficiency of heMSC-microcarrier constructs was performed using commercially available, dextran-based, positively-charged Cytodex 1 microcarriers (Fig. 1). To this end, heMSC were cultivated on Cytodex 1 microcarriers for 7 days in an agitated spinner culture (Fig. 1a). heMSC growth kinetics on Cytodex 1 microcarriers showed the attainment of an early-logarithmic phase with 43% cell confluency at day 3, a mid-logarithmic phase with 68% cell confluency at day 5, and a late-logarithmic phase with 95% cell confluency at day 7 of microcarrier-spinner culture (Fig. 1a).

For the first stage of the screening study, cell confluency and cell numbers per construct were tested (Fig. 1b). heMSC-covered microcarriers either with 43% cell confluency (day 3), or with 68% cell confluency (day 5), or with 95% cell confluency (day 7) were used to generate a total of 12 distinct constructs containing either 2, 10, 50, or 200 × 10^3^ cells per construct (Fig. 1b). The combinations of different cell confluencies, cell numbers per construct, and resultant microcarrier numbers per construct are presented in Table 2. After chondrogenic differentiation for 21 days, these heMSC-Cytodex 1 constructs were evaluated based on two distinct criteria: 1) cell growth by measuring total DNA per construct; and 2) chondrogenic output by measuring total GAG and Collagen II per construct (Fig. 1b).

**Table 2:**
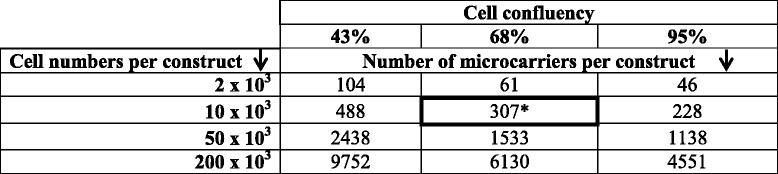
Number of microcarriers per construct used to generate a 12-combination matrix of various cell confluencies and cell numbers per heMSC-Cytodex 1 constructs

^*^Optimally defined conditions: ~70% cell confluency, 10 × 10^3^ cells per construct, ~300 microcarriers per construct, ~33 cells per microcarrier

**Fig. 2:**
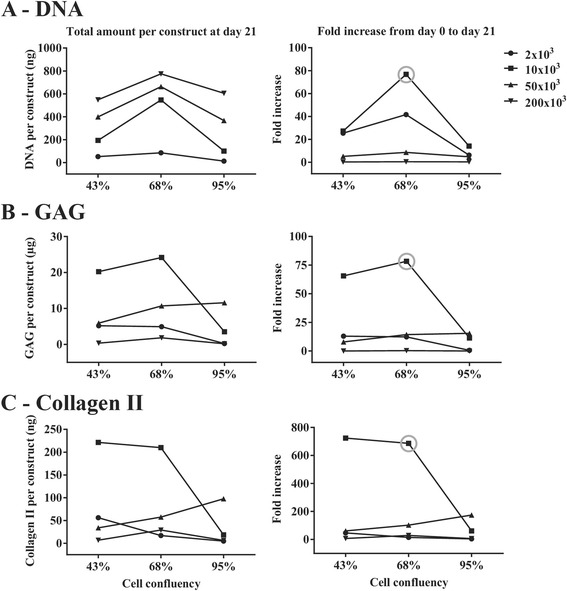
Seeding 10 × 10^3^ cells at 68% cell confluency per heMSC-Cytodex 1 construct (*grey circle*) resulted in efficient cell growth and chondrogenic differentiation by 21 days of differentiation. **a** DNA, **b** GAG, and **c** Collagen II content per construct by day 21 of differentiation as well as respective fold-increases from day 0 to day 21 of differentiation

The most efficient cell growth and chondrogenic differentiation was achieved at 68% cell confluency with 10 × 10^3^ cells and about 307 microcarriers per construct (grey circle in Fig. 2 and bold box in Table 2). This critically defined heMSC-Cytodex 1 construct produced a considerable amount of DNA (546 ng), the highest amount of GAG (24.2 μg), and a substantial amount of Collagen II (210 ng) per construct (Fig. 2a–c). Most importantly, it achieved the greatest fold-increase in DNA (76.8-fold) and GAG (78.3-fold) content per construct with the second greatest fold-increase in Collagen II (686-fold) content per construct from day 0 to day 21 of differentiation, as compared to all other constructs (Fig. 2a–c).

Cell growth and chondrogenic differentiation were affected by both cell confluency and cell numbers per construct (Fig. 2a). Seeding heMSC-microcarrier constructs at a high cell confluency of 95% generally produced lower amounts of DNA, GAG, and Collagen II and induced lower fold-increases in DNA, GAG, and Collagen II content per construct across all cell numbers when compared to that at 68% cell confluency (Fig. 2a–c). Seeding heMSC-microcarrier constructs at low and high cell numbers, namely 2, 50, and 200 × 10^3^ cells per construct, produced lower amounts of GAG and Collagen II and induced lower fold-increases in DNA, GAG, and Collagen II content per construct across all cell confluencies as compared to that at 10 × 10^3^ cells per construct (Fig. 2a–c). It is important to note that cell growth and chondrogenic protein production is not linked. For instance, heMSC-Cytodex 1 construct seeded with 10 × 10^3^ cells per construct at 43% cell confluency displayed low fold-increase in DNA content but achieved the second highest fold-increase in GAG and Collagen II production (Fig. 2a–c). In contrast, heMSC-Cytodex 1 construct seeded with 2 × 10^3^ cells per construct at 68% cell confluency displayed the second highest fold-increase in DNA content but achieved low levels of fold-increase in GAG and Collagen II production (Fig. 2a–c).

At the second stage of the screening study, the effect of cell density and compaction of the construct by either centrifugation at seeding or agitation throughout differentiation were investigated using the optimal parameters defined at the first stage (Fig. 1b).

**Fig. 3:**
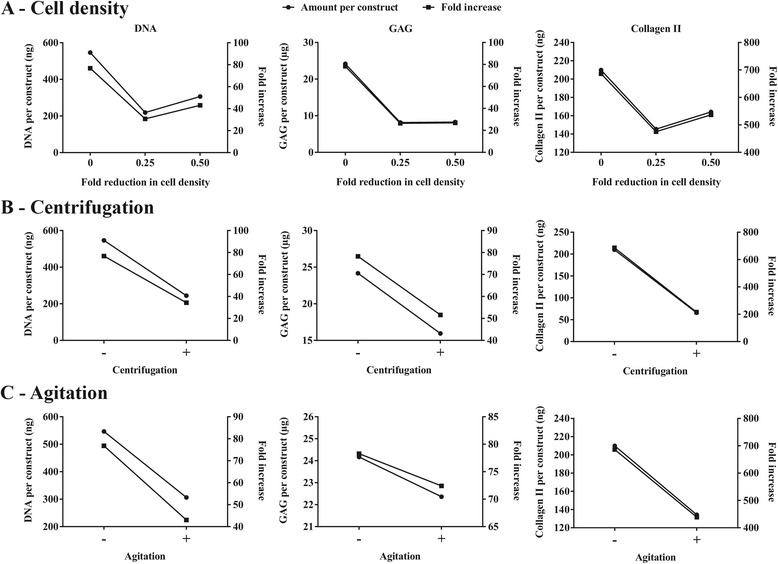
Reduction in cell density by adding empty microcarriers at seeding and construct compaction by applying centrifugation at seeding or continuous agitation throughout differentiation had a negative impact on cell growth and chondrogenic output. **a** DNA, **b** GAG, and **c** Collagen II content per construct at day 21 of differentiation and relevant fold-increases from day 0 to day 21 of differentiation

The change in cell density was achieved by adding either 0%, 25%, or 50% more empty microcarriers to the critically defined heMSC-Cytodex 1 constructs (10 × 10^3^ cells, 70% cell confluency, 300 microcarriers per construct) at seeding. As described in Table 3, the addition of 25% and 50% more microcarriers resulted in a reduction of cell density (cells per mg of microcarriers) by 0.25- and 0.50-fold, respectively, and led to an increase in cell aggregate size (number of microcarriers per construct) without affecting cell numbers per construct at seeding or day 0 of differentiation. Results presented in Fig. 3a showed that the addition of 25% and 50% more microcarriers resulted in a decrease in cell growth and chondrogenic output, as evident by reductions in DNA (60.0% and 44.0% reductions, respectively), GAG (66.3% and 65.8% reductions, respectively), and Collagen II (30.7% and 21.8% reductions, respectively) production when compared with that of constructs with a 0% addition of microcarriers. This decrease in cell growth and chondrogenic differentiation efficiency can be attributed to the lowering of cell concentration or the increase in aggregate size at seeding (Table 3).

**Table 3:** Effect of the addition of empty microcarriers at seeding on cell density (number of cells per microcarrier and per cm^2^) as well as aggregate size (number of microcarriers per construct)

Fold reduction in cell density	0^a^	0.25	0.50
Cells per construct	10 × 10^3^	10 × 10^3^	10 × 10^3^
Cytodex 1 microcarriers (mg) per construct	0.0713	0.0886	0.1069
Cells per mg of microcarriers (cells/mg)	1.40 × 10^5^	1.13 × 10^5^	9.35 × 10^4^
Cytodex 1 microcarriers (number) per construct	307	381	460
Cells per microcarrier (cells)	32.6	26.3	21.8
Surface area of Cytodex 1 microcarriers (cm^2^) per construct	0.314	0.390	0.470
Cells per cm^2^ (cells/cm^2^)	31,885	25,663	21,256

^a^Optimally defined conditions: 70% cell confluency, 10 × 10^3^ cells per construct, ~300 microcarriers per construct, ~33 cells per microcarrier

Compaction of the critically defined heMSC-Cytodex 1 constructs was achieved by either centrifugation of the constructs at 1000 rpm for 5 min at seeding, or continuous agitation of the constructs at 100 rpm throughout differentiation. Applying these treatments resulted in a decrease in cell growth and chondrogenic output when compared to that of untreated constructs, as evident by reductions in DNA (55.3% and 44.0% reductions, respectively), GAG (34.1% and 7.49% reductions, respectively), and Collagen II (68.8% and 36.0% reductions, respectively) production (Fig. 3b and c). This decrease in cell growth and chondrogenic differentiation efficiency can be caused by the mechanical stress applied.

In conclusion, this study identified three out of the five parameters tested, namely cell confluency, cell numbers per construct, and microcarrier numbers per construct, to be critical in enabling effective cell growth and chondrogenic differentiation of heMSC-Cytodex 1 constructs (Figs. 2 and 3). The robustness of the identified parameters were tested by repeating the experiments generating six critically defined heMSC-Cytodex 1 constructs where we achieved similar cell growth and chondrogenic output per construct with a narrow range of deviations (DNA: 464 ± 112 ng; GAG: 19.8 ± 7.85 μg; Collagen II: 157 ± 49.8 ng). Our results showed that a narrow range of cell confluency (about 70%), cell number (10 × 10^3^ cells), and microcarrier number (about 300) per construct were necessary to generate the optimal microenvironment for efficient chondrogenic differentiation of heMSC-laden microcarrier constructs (Figs. 2 and 3; Tables 1–3). These critically defined parameters were used in the following studies.

### Comparison of cell growth and chondrogenic differentiation efficiency in critically defined heMSC-microcarrier constructs to equivalent cell-only pellets

The next question was whether continuous cell attachment to microcarriers throughout differentiation in critically defined heMSC-microcarrier constructs would also display improved cell growth and chondrogenic differentiation as compared to equivalent cell-only chondrogenic pellets derived from conventional monolayer cultures on tissue culture plastic. To this end, cell growth kinetics, the chondrogenic differentiation process, and histological studies of critically defined heMSC-Cytodex 1 constructs seeded with 10 × 10^3^ cells at about 70% cell confluency with about 300 microcarriers per construct as well as cell-only chondrogenic pellets seeded with 10 × 10^3^ cells at about 70% cell confluency (derived from tissue culture plastic) were performed and compared.

**Fig. 4:**
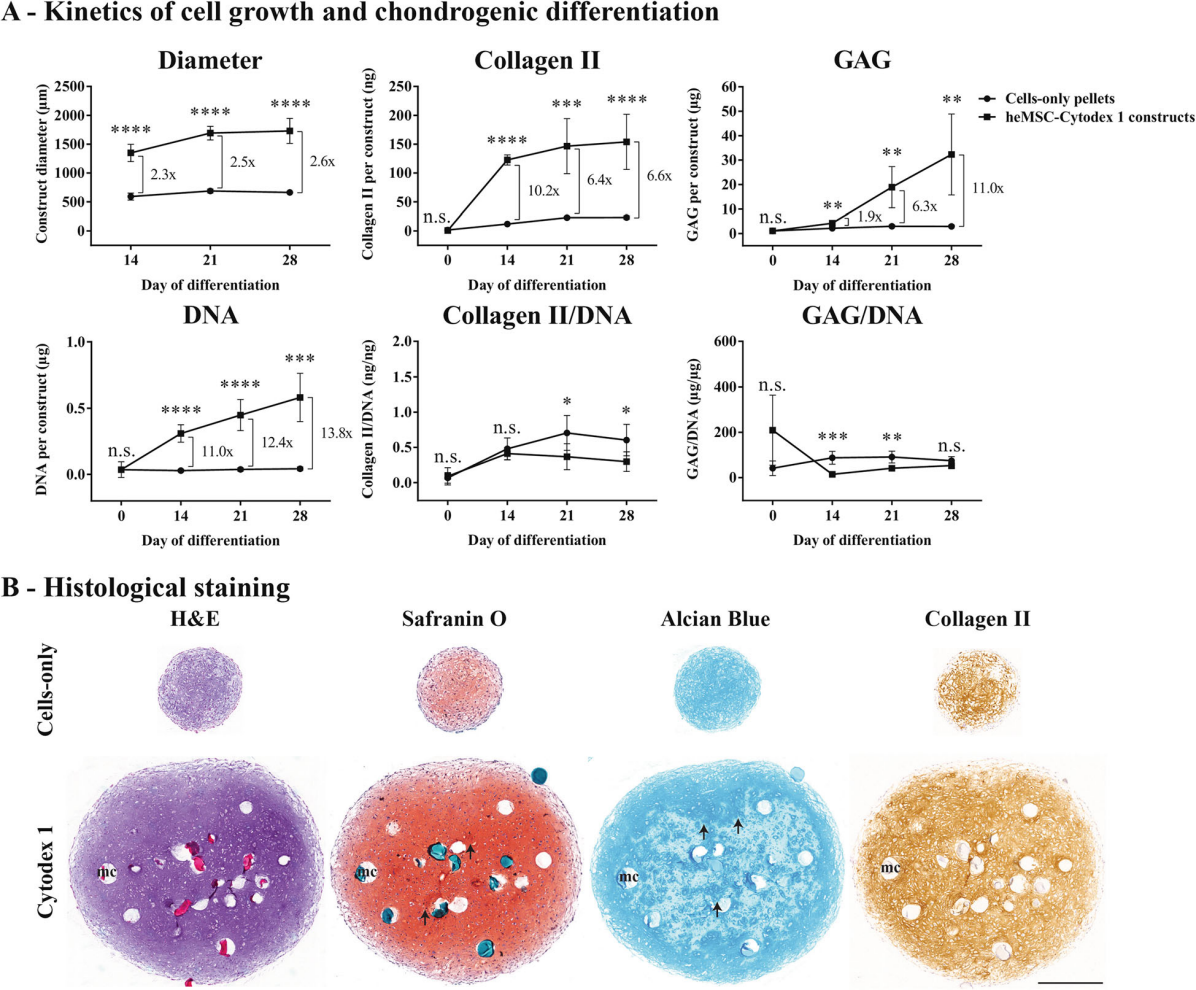
heMSC-Cytodex 1 constructs developed larger pellet diameters, increased cellular proliferation, and, most importantly, improved total chondrogenic output in terms of proteoglycan and Collagen II content as compared to their equivalent cell-only counterparts. **a** Kinetics of cell growth and chondrogenic differentiation. heMSC-Cytodex 1 constructs and cell-only pellets were seeded with 10 × 10^3^ heMSC at 70% cell confluency. Kinetics of construct/pellet diameter, DNA, GAG, and Collagen II production were monitored during 28 days of differentiation. All *p* values refer to statistical significance obtained by comparing heMSC-Cytodex 1 constructs to that of cell-only counterparts at the indicated time points. *p* values: n.s. = *p* > 0.05, *p < 0.05, **p < 0.01, ***p < 0.001. and ****p < 0.0001. All numbers shown indicate the fold-changes of heMSC-Cytodex 1 constructs over that of cell-only pellets at the indicated time points. **b** Histological H&E, Safranin O, Alcian Blue, and Collagen II staining of heMSC-Cytodex 1 constructs and cell-only pellets at day 28 of differentiation. *Arrows* indicate areas in heMSC-Cytodex 1 constructs with more intense staining compared to that of cell-only pellets. The space occupied by the microcarrier is indicated as “mc”. *Scale bar* = 500 μm

Results presented in Fig. 4a show that critically defined heMSC-Cytodex 1 constructs displayed enhanced cell growth compared to cell-only pellets (1.28-fold as compared to 1.12-fold increase in diameter from day 14 to day 28, and 16.6-fold as compared to 1.24-fold increase in DNA from day 0 to day 28). By day 28 of differentiation, heMSC-Cytodex 1 constructs had a 2.6-fold bigger diameter (*p* < 0.0001) and 13.8-fold more DNA (*p* = 0.0002) than that of cell-only pellets (Fig. 4a).

Results presented in Fig. 4a also revealed that critically defined heMSC-Cytodex 1 constructs displayed improved chondrogenic output per construct as they achieved a 184-fold and 29.8-fold increase in Collagen II and GAG, respectively, from day 0 to day 28 while cell-only pellets attained only a 14.3-fold and 2.61-fold increase in Collagen II and GAG, respectively, from day 0 to day 28. By day 28 of differentiation, heMSC-Cytodex 1 constructs had produced 6.6-fold more Collagen II (*p* < 0.0001) and 11.0-fold more GAG (*p* = 0.0042) than cell-only pellets (Fig. 4a). This increase in Collagen II and GAG production was mainly due to the increase in cell number but not to the increase in chondrogenic protein production per cell as the Collagen/DNA and GAG/DNA ratios were similar between the heMSC-Cytodex 1 constructs and control cell-only pellets (Fig. 4a). This suggests that cell attachment to microcarriers throughout differentiation is a critical factor that can improve total chondrogenic output primarily by enhancing cellular proliferation.

H&E staining from day 14 through day 28 of differentiation showed that heMSC-Cytodex 1 constructs grew larger than their cell-only counterparts (Fig. 4b; data shown for day 28). This increase in size was not only due to the presence of microcarriers but was also caused by an increase in the number of cells (Fig. 4b; data shown for day 28). Safranin O and Alcian Blue staining for proteoglycan, including GAG, content revealed that heMSC-Cytodex 1 constructs not only developed more intense staining (arrows) but also had a larger proportion of the construct positively stained as compared to that of cell-only pellets from day 14 to day 28 of differentiation (Fig. 4b; data shown for day 28). Immunocytochemical staining for Collagen II also showed that these constructs had nearly all of the construct cross-section positively stained for Collagen II while their cell-only counterparts had many discrete areas around the periphery that were negative for Collagen II (Fig. 4b; data shown for day 28).

### Comparison of cell growth and chondrogenic output of heMSC-microcarrier constructs generated using cell-covered microcarriers obtained from the 5-day expansion process in microcarrier-spinner cultures or the 1-day attachment process in shake flask cultures

**Fig. 5:**
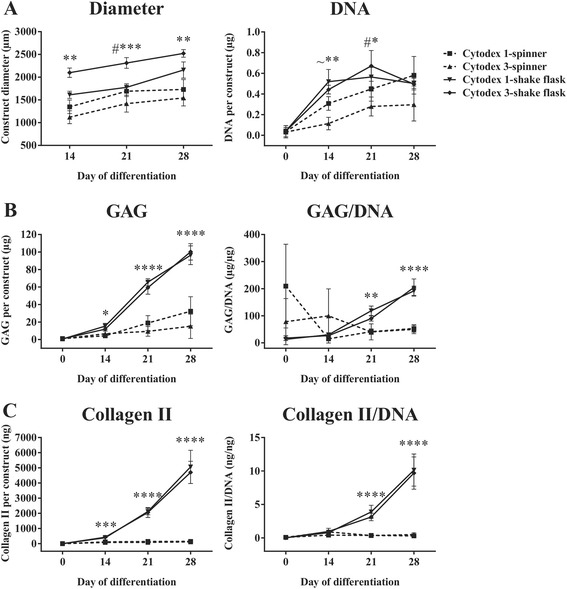
heMSC-Cytodex 1/3 constructs created via agitated shake flask platform were more efficient in promoting cell growth as well as chondrogenic output per construct and per cell as compared to those derived from agitated spinner platform. **a** Construct diameter and DNA content per construct. **b** GAG content per construct and GAG/DNA ratio. **c** Collagen II content per construct and Collagen II/DNA ratio. *p* values: *p < 0.05, **p < 0.01, ***p < 0.001, and ****p < 0.0001. All *p* values refer to the statistical significance of heMSC-Cytodex 1/3 constructs from agitated shake flask platform compared to those derived from spinner platform at the indicated time points. ^#^All comparisons except between Cytodex 1-shake flask and Cytodex 1-spinner. ^~^All comparisons except between Cytodex 3-shake flask and Cytodex 1-spinner

To investigate whether the mode of attaching cells to microcarriers and the type of agitation have an effect on cell growth and chondrogenic differentiation outcomes, we generated critically defined heMSC-Cytodex 1 and heMSC-Cytodex 3 constructs using cell-laden microcarriers generated by a 5-day cell expansion process in an agitated spinner culture or by a 1-day cell attachment process in an agitated shake flask culture, and compared their cell growth and chondrogenic output through 28 days of differentiation (Fig. 5). All heMSC-Cytodex 1/3 constructs were generated from 300 cell-laden microcarriers having 70% cell confluency. In the spinner cultures, 70% cell confluency was achieved within 5 days of growth while, in the shake flask cultures, cells harvested from monolayer cultures on tissue culture plastic were added in amounts assuring 70% confluency with more than 95% of the cells attaching to the microcarriers within a day (Table 1).

Results presented in Fig. 5a show that heMSC-Cytodex 1/3 constructs derived from shake flask cultures (solid lines) enabled significantly higher cell growth as they developed larger construct diameters (day 14: *p* = 0.0086; day 21: *p* = 0.0007; day 28: *p* = 0.0044), and produced more DNA (day 14: *p* = 0.0028; day 21: *p* = 0.0332) when compared to those derived from spinner cultures (dotted lines). Moreover, heMSC-Cytodex 1/3 constructs derived from shake flask cultures also significantly improved GAG (day 14: *p* = 0.0236; day 21 and day 28: *p* < 0.0001) and Collagen II (day 14: *p* = 0.0002; day 21 and day 28: *p* < 0.0001) production per construct as early as day 14 through day 28 of differentiation, as well as GAG (day 21: *p* = 0.0034; day 28: *p* < 0.0001) and Collagen II (all *p* < 0.0001) production per cell from day 21 through day 28 of differentiation, when compared to those derived from spinner cultures (Fig. 5b and c). Thus, our results showed that heMSC-microcarrier constructs derived from the 1-day cell attachment process in the shake flask platform performed better than the ones generated from the 5-day expansion process in spinner flasks. This enhancement results from both improving cell growth as well as increasing GAG and Collagen II production per cell. These results suggest that the state of cells (freshly attached for 1 day or expanded for 5 days) and mode of agitation (1 day shake flask or 5 days in stirred spinner flask) before the differentiation process may have an effect on cell growth and chondrogenic output.

### Applying critically defined parameters across multiple microcarrier types

To test whether the critical attributes that we have identified can be applied across different microcarrier types, as well as to ascertain whether the improvement in cell growth and chondrogenic output by continuous cell attachment to microcarriers throughout differentiation can be achieved with other microcarrier types, we created distinct heMSC-microcarrier constructs using cell-laden Cytodex 1, Cytodex 3, SphereCol, and Cultispher-S microcarriers from an agitated shake flask culture, and compared their cell growth and chondrogenic output with that of cell-only chondrogenic pellets derived from conventional tissue culture plastic (10 × 10^3^ cells at 70% cell confluency per pellet without any prior attachment to microcarriers) for 28 days of differentiation. A summary describing the characteristics of the different microcarriers tested and their respective seeding conditions at day 0 of differentiation can be found in Table 1. It is important to note that these microcarriers have different sizes (ranging from 100 to 400 μm), matrices (dextran or collagen), surface nature (positively charged or denatured collagen), and surface appearance (smooth or porous) (Table 1). Two out of the four microcarrier types are collagen-based, thus they are biodegradable (Table 1). All of the microcarriers except Cultispher-S are similar in size and are microporous with a smooth surface where cells would only attach to the outer surface of the carrier. On the other hand, Cultispher-S microcarriers are larger, have nonsmooth surface and are macroporous in nature where cells can grow not only on the microcarrier surface but also within the internal microcarrier space, thus making it difficult to define critical parameters for Cultispher-S microcarriers.

**Fig. 6:**
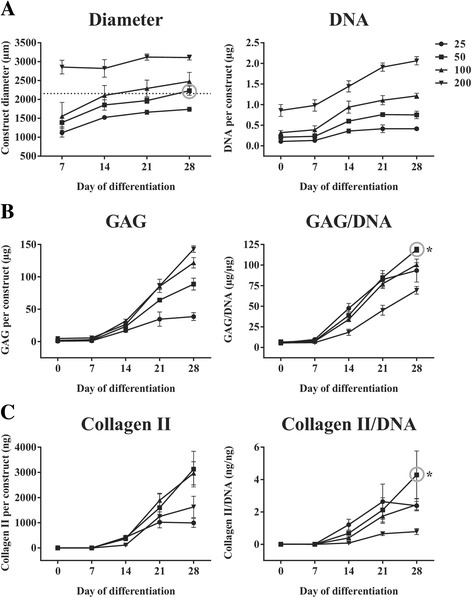
heMSC-Cultispher-S constructs seeded with 50 microcarriers per construct with 70% cell confluency induced effective cell growth and chondrogenic differentiation. **a** Construct diameter and DNA content per construct. *Dotted line* indicates construct diameter attained by optimally defined heMSC-Cytodex 1 constructs after 28 days of differentiation. This diameter is obtained by heMSC-Cultispher-S constructs seeded with 50 microcarriers per construct (*grey circle*). **b**, **c** Glycosaminogycan (*GAG*) and Collagen II content per construct as well as GAG/DNA and Collagen II/DNA ratios. heMSC-Cultispher-S constructs seeded with 50 microcarriers (*grey circle*) significantly induced the highest GAG/DNA and Collagen II/DNA ratios at day 28 of differentiation when compared to constructs with different microcarrier numbers. *p* values: *p < 0.05

In order to define the optimal parameters to cultivate heMSC-Cultispher-S constructs for efficient chondrogenic differentiation, we first produced 70% confluent cell-covered Cultispher-S microcarriers (based on the microcarrier surface area, as provided by the manufacturer) using the 1-day cell attachment process in agitated shake flask cultures. Then heMSC-Cultispher-S constructs containing 25, 50, 100, or 200 microcarriers per construct were generated and differentiated along the chondrogenic lineage for 28 days (Fig. 6). Comparative analysis showed that optimal conditions were achieved with 50 Cultispher-S microcarriers per construct, as these constructs (grey circle) developed similar diameters as those of critically defined heMSC-Cytodex 1 constructs (dotted line, 2160 μm) by day 28 of differentiation (Fig. 6a), and significantly produced more GAG per μg DNA (*p* = 0.0126) and Collagen II per μg DNA (*p* = 0.0141) at day 28 of differentiation as compared with the other heMSC-Cutltispher-S constructs (Fig. 6b and c).

With all other microcarrier types, we seeded heMSC with the equivalent amount of cells to achieve 70% confluency per type of microcarrier bead, using the 1-day cell attachment process in agitated shake flask cultures. Within a day, when more than 95% of the cells were attached to the microcarriers, we created heMSC-Cytodex 1, Cytodex 3, and SphereCol constructs with 300 microcarriers per construct. Details on the seeding conditions per heMSC-microcarrier construct across the distinct microcarrier types can be found in Table 1.

**Fig. 7:**
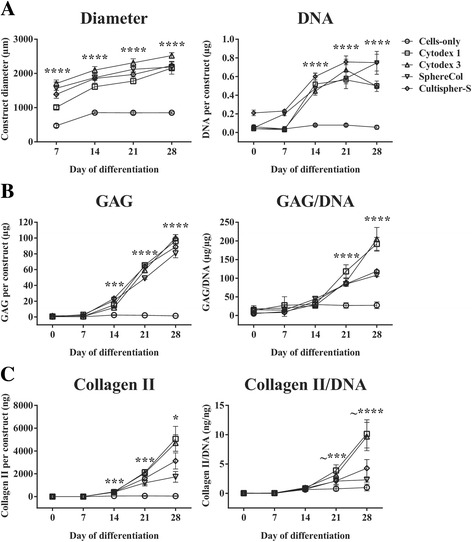
heMSC-microcarrier constructs of different microcarrier types increased cellular proliferation and improved chondrogenic output per construct and per cell when compared to their equivalent cell-only counterparts. **a** Construct diameter and DNA content per construct. **b** Glycosaminogycan (*GAG*) content per construct and GAG/DNA ratio. **c** Collagen II content per construct and Collagen II/DNA ratio. *p* values, *p < 0.05, ***p < 0.001, and ****p < 0.0001. All *p* values refer to the statistical significance of all heMSC-microcarrier constructs across distinct microcarrier types over that of cell-only counterparts at the indicated time points. ^~^All heMSC-microcarrier pellets except that of SphereCol and Cultispher-S

**Fig. 8:**
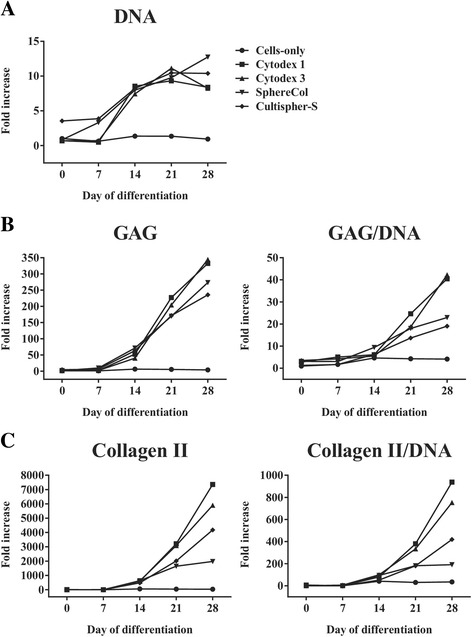
heMSC-microcarrier constructs of different microcarrier types increased cellular proliferation and improved chondrogenic output per construct and per cell when compared to their equivalent cell-only counterparts. Fold-increases of **a** DNA content per construct, **b** Glycosaminogycan (*GAG*) content per construct and GAG/DNA ratio, and **c** Collagen II content per construct and Collagen II/DNA ratio across different microcarrier types over the cell-only value at day 0 of differentiation

Comparing the cell growth kinetics and chondrogenic output of heMSC-Cytodex 1, heMSC-Cytodex 3, heMSC-SphereCol, and heMSC-Cultispher-S constructs (50 microcarriers per construct) to cell-only chondrogenic pellets, we found that heMSC-microcarrier constructs across multiple microcarrier types significantly enhanced cell growth as they developed larger construct diameters (all *p* < 0.0001) as early as day 7 through day 28 of differentiation, and produced more DNA (all *p* < 0.0001) from day 14 through day 28 of differentiation, when compared to that of cell-only pellets (Fig. 7a). Moreover, these constructs across different microcarrier types also significantly improved chondrogenic output by enhancing GAG (day 14: *p* = 0.0006; day 21 and day 28: *p* < 0.0001) and Collagen II (day 14: *p* = 0.0001; day 21: *p* = 0.0003; day 28: *p* = 0.0196) production per construct as early as day 14 through day 28 of differentiation (Fig. 7b and c). Most importantly, they also improved GAG (all *p* < 0.0001) and Collagen II (day 21: *p* = 0.0005 for Cytodex 1 and 3; day 28: *p* < 0.0001 for Cytodex 1 and 3) production per cell from day 21 through day 28 of differentiation, when compared to that of cell-only pellets (Fig. 7b and c). Fold-increases achieved by the distinct heMSC-microcarrier constructs over that of day 0 cell-only pellets can be found in Fig. 8.

To confirm our findings and determine whether heMSC-microcarrier constructs across multiple microcarrier types display improved chondrogenic output at a transcriptome level as well, we compared the chondrogenic gene expression profiles of critically defined heMSC-microcarrier constructs across 28 days of differentiation to that of cell-only pellets. At day 0 of differentiation, we found that critically defined heMSC-microcarrier constructs showed similar expression levels across the repertoire of chondrogenic and hypertrophic markers tested when compared to cell-only pellets (Additional file 1: Table S2). In contrast, at day 28 of differentiation, these critically defined heMSC-microcarrier constructs displayed not only a significant upregulation of chondrogenic markers, but also a significant downregulation of hypertrophic markers such as MMP13 as compared to their cell-only counterparts (Additional file 1: Table S2). This further supports our findings that critically defined heMSC-microcarrier constructs across multiple microcarrier types improve chondrogenic differentiation outcomes, and is in line with the biochemical data presented in Figs. 7 and 8.

Since the enhancement in cell growth and chondrogenic output is seen across the different microcarrier types with different surface characteristics (ionic charges or protein coating), it can be assumed that the three-dimensional configuration of the microcarriers may be more important than the surface characteristics in influencing the improvement. To ascertain this, we compared the cell growth and chondrogenic output of cell-only pellets derived from gelatin-coated and noncoated two-dimensional tissue culture plastic. We found no significant differences between the DNA, GAG, and Collagen II production per construct and per cell at days 21 and 28 of differentiation between these two conditions (Additional file 1: Figure S2). Hence, these results further support the observation that the three-dimensional configuration of the microcarriers, as opposed to the two-dimensional structure of tissue culture plastic, is likely to be more influential in enhancing chondrogenic differentiation of heMSC.

Interestingly, while constructs made up of collagen/gelatin-based microcarriers (e.g., SphereCol and Cultispher-S) produced similar amounts of GAG per construct, they induced lower amounts of GAG per cell, as well as Collagen II per construct and per cell, when compared to that of constructs made up of dextran-based microcarriers (e.g., Cytodex 1 and Cytodex 3) (Fig. 7b and c). This suggests that matrix material can affect chondrogenic differentiation outcomes.

In conclusion, the identified critical parameters described for Cytodex 1 microcarriers are applicable across different microcarrier types. Most importantly, our findings demonstrate that cell attachment to the microcarrier throughout differentiation across multiple microcarrier types in critically defined heMSC-microcarrier constructs can improve cell growth as well as total chondrogenic output per construct and per cell when compared to that of cell-only pellets. It is important to note that cell growth and chondrogenic output can change to some degree between different types of microcarriers, indicating that further refinement of the critical attributes specific to each microcarrier type may be required.

## Discussion

The first attempt to differentiate hMSC-seeded microcarriers as modular constructs for cartilage tissue engineering was made by Georgi et al. [34]. In their work, they reported an approximate two- to threefold increase in total DNA content of their hMSC-seeded microcarrier construct after 4 weeks of culture when compared to their hMSC single-cell construct without microcarriers [34]. While this is in general agreement with our results, we obtained much higher fold-increases in DNA content per construct that range from as low as 8.6-fold for Cytodex 3 microcarriers to 13.1-fold for SphereCol microcarriers by 28 days of differentiation (Figs. 4, 7, and 8). Although this could be due to differences in the hMSC lines used, it is likely that the improvement in cell growth rates is due to the successful identification of the critical parameters needed to grow hMSC-microcarrier constructs efficiently.

In contrast, Georgi et al. showed that their hMSC-seeded microcarrier construct did not differentiate as well as their single-cell construct, with a two- to threefold decrease in GAG/DNA ratio and nonuniform GAG deposition only in the bottom but not upper and middle layers of the construct, as evident by Alcian Blue staining [34]. This differs with our results where we showed an increase not only in GAG production per construct and per cell, but also an increase in Collagen II production per construct and per cell across hMSC-microcarrier constructs of different microcarrier types (Figs. 4, 7, and 8). Furthermore, the hMSC-microcarrier constructs showed rather uniform chondrogenic differentiation all around the construct as evident by proteoglycan and Collagen II deposition (Fig. 4). This disparity could be due to the difference in size between the critically defined heMSC-microcarrier constructs (less than 3000 μm in diameter even after 28 days of cell growth and differentiation) to theirs (more than 6 mm in diameter and 4 mm in height at seeding), which may affect differentiation outcomes due to diffusion limitations of nutrient exchange. As our screening study had shown, increasing the number of microcarriers per construct had a detrimental effect on cell growth and chondrogenic output in terms of GAG and Collagen II production (Fig. 3). Thus, there is a critical construct size that enables efficient chondrogenic differentiation of hMSC-microcarrier constructs; exceeding this size will decrease differentiation efficiency.

Interestingly, our results show that heMSC-microcarrier constructs derived from an agitated shake flask platform developed better cell growth and chondrogenic differentiation outcomes as compared to constructs derived from an agitated spinner platform (Fig. 5). This difference may be caused by dissimilarities in agitation method (rotation-based in shake flask cultures versus impeller-based in spinner cultures), or by the difference between freshly attached cells (1-day cell attachment to microcarriers with no further cell growth in shake flask cultures) and cells expanded on the microcarriers (5 days of cell growth on microcarriers in spinner cultures).

heMSC-microcarrier constructs with dextran-based microcarriers (Cytodex 1 and 3) performed better in enhancing chondrogenic output, particularly in Collagen II output per construct and per cell, than constructs with collagen/gelatin-based microcarriers (SphereCol and Cultispher-S) (Figs. 7 and 8). This suggests that matrix material or even matrix stiffness may affect differentiation outcomes. On the other hand, given that Cytodex 1 (positively charged) and Cytodex 3 (gelatin-coated) achieve similar levels of cell growth and chondrogenic output, it suggests that microcarrier coating or charges is unlikely to be as crucial in determining differentiation outcomes (Figs. 7 and 8). This is further supported when we observed no significant differences in terms of cell growth and chondrogenic output when we compared cell-only chondrogenic pellets derived from either gelatin-coated or noncoated two-dimensional tissue culture plastic, which suggest that the gelatin coating alone did not improve cell growth and differentiation outcomes (Additional file 1: Figure S2).

## Conclusions

This study is the first to successfully identify a set of critical parameters, namely cell confluency, cell numbers per construct, and microcarrier numbers per construct, to be important for enabling efficient chondrogenic differentiation of heMSC-microcarrier constructs across multiple microcarrier types including Cytodex 1, Cytodex 3, SphereCol, and Cultispher-S. We show that the critically defined hMSC-microcarrier constructs require at least 20-times fewer cells for seeding at 10 × 10^3^ cells than that of conventional cell-only chondrogenic pellets that would require 200 × 10^3^ cells, which is greatly advantageous as it allows greater economies of scale. Most importantly, we demonstrate that continuous cell attachment to microcarriers throughout differentiation using the critically defined MSC-microcarrier constructs with different microcarrier types improved cell growth and chondrogenic output per construct and per cell when compared to that of equivalent cell-only pellets. This shows that using microcarriers as scaffold material in heMSC-microcarrier constructs confers many advantages, and further supports their use not only for large-scale cell expansion but also for creating hMSC-microcarrier chondrogenic constructs that can be used modularly for therapeutic applications in allogeneic cartilage cell and tissue therapy.

## Additional file

Additional file 1: Figure S1. Histological stainings in H&E, Safranin O, and Alcian Blue as well as Collagen II immunostaining of native rabbit cartilage. Scale bar = 200 μm. **Figure S2.** Cell-only chondrogenic pellets derived from either gelatin-coated or noncoated two-dimensional tissue culture plastic displayed similar levels of cell growth and chondrogenic output per construct and per cell. (A) DNA content per pellet. (B) GAG content per pellet and GAG/DNA ratio. (C) Collagen II content per pellet and Collagen II/DNA ratio. *p* values: n.s. = nonsignificant. All *p* values refer to the statistical significance of gelatin-coated pellets over that of noncoated counterparts at the indicated time points. All numbers shown indicate the fold-changes of gelatin-coated pellets over that of noncoated counterparts at the indicated time points. **Table S1.** List of qRT-PCR Taqman® probes used in this study. **Table S2.** Gene expression profiles of critically defined heMSC-microcarrier constructs across different microcarrier types at day 0 and 28 of differentiation. All numbers shown indicate the fold-changes of heMSC-microcarrier constructs over that of the cell-only counterparts at the indicated time points. (DOCX 2425 kb)

## Abbreviations

GAG: Glycosaminoglycan
heMSC: Human early mesenchymal stromal cells
hMSC: Human mesenchymal stromal cells
MSC: Mesenchymal stromal cells
