# Supplementary material for: Critical attributes of human early mesenchymal stromal cell-laden microcarrier constructs for improved chondrogenic differentiation
Source: Stem Cell Res Ther. 2017 May 8;8:93. doi: 10.1186/s13287-017-0538-x (PMC5421335; doi:10.1186/s13287-017-0538-x)
Supplement: Additional file 1: Figure S1. — Histological stainings in H&E, Safranin O, and Alcian Blue as well as Collagen II immunostaining of native rabbit cartilage. Scale bar = 200 μm. Figure S2. Cell-only chondrogenic pellets derived from either gelatin-coated or noncoated two-dimensional tissue culture plastic displayed similar levels of cell growth and chondrogenic output per construct and per cell. (A) DNA content per pellet. (B) GAG content per pellet and GAG/DNA ratio. (C) Collagen II content per pellet and Collagen II/DNA ratio. p values: n.s. = nonsignificant. All p values refer to the statistical significance of gelatin-coated pellets over that of noncoated counterparts at the indicated time points. All numbers shown indicate the fold-changes of gelatin-coated pellets over that of noncoated counterparts at the indicated time points. Table S1. List of qRT-PCR Taqman® probes used in this study. Table S2. Gene expression profiles of critically defined heMSC-microcarrier constructs across different microcarrier types at day 0 and 28 of differentiation. All numbers shown indicate the fold-changes of heMSC-microcarrier constructs over that of the cell-only counterparts at the indicated time points. (DOCX 2425 kb) [file 13287_2017_538_MOESM1_ESM.docx]

**Lin.Sup.Fig.1.**

**
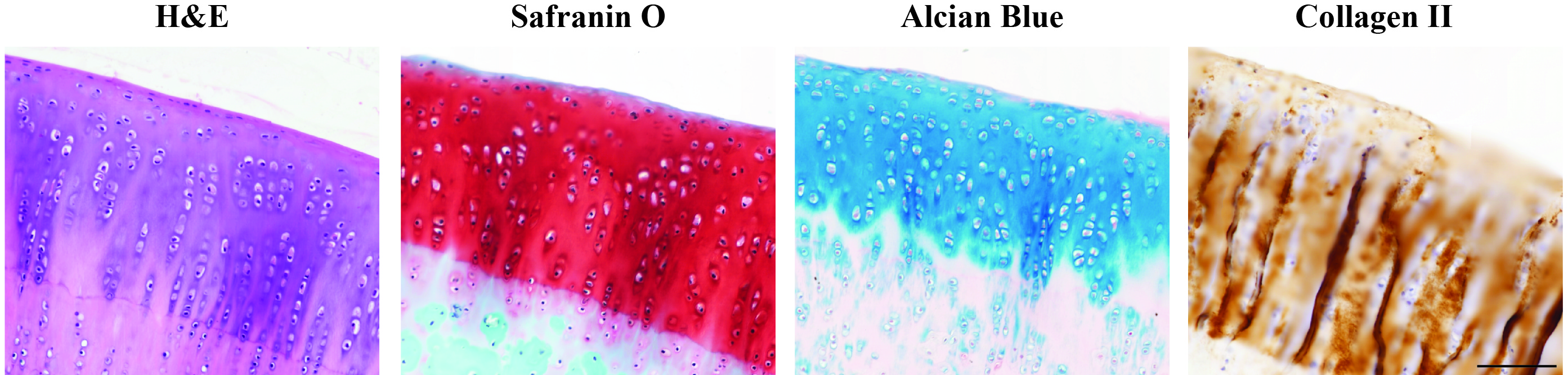
Sup.Fig.1.** Histological stainings in H&E, Safranin O and Alcian Blue as well as Collagen II immunostaining of native rabbit cartilage. Scale bar, 200 μm.

**Lin.Sup.Fig.2.**

**
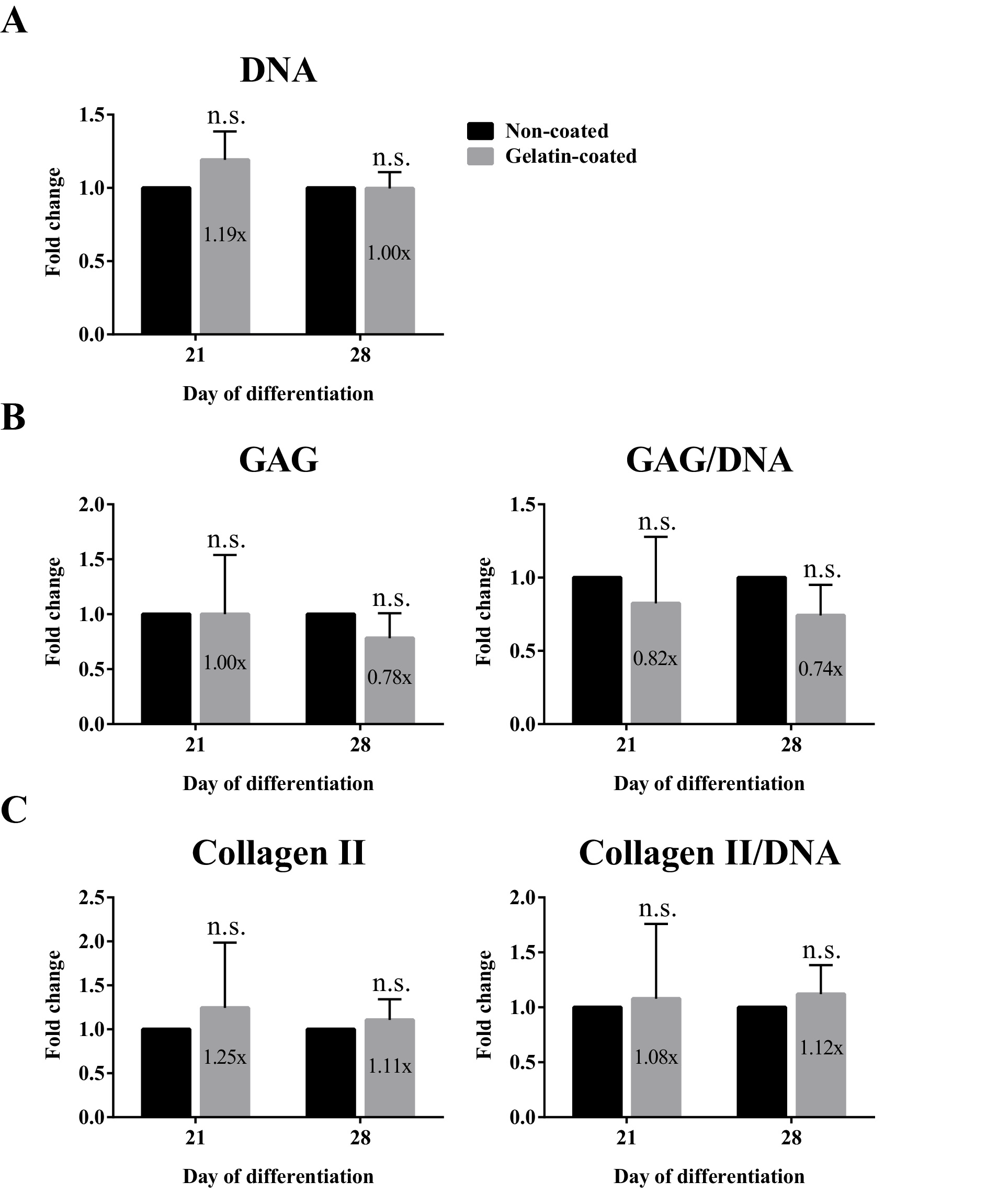
**

**Sup.Fig.2.** Cells-only chondrogenic pellets derived from either gelatin-coated or non-coated 2D tissue culture plastic displayed similar levels of cell growth and chondrogenic output per construct and per cell. **(A)** DNA content per pellet. **(B)** GAG content per pellet and GAG/DNA ratio. **(C)** Collagen II content per pellet and Collagen II/DNA ratio. p values, n.s. = non-significant. All p values refer to the statistical significance of gelatin-coated pellets over that of non-coated counterparts at indicated timepoints. All numbers shown indicate the fold changes of gelatin-coated pellets over that of non-coated counterparts at indicated timepoints.

**Lin.Sup.Table.1**

| **Gene** | **Catalog number** |
| --- | --- |
| Human SOX9 | Hs01001343_g1 |
| Human L-SOX5 | Hs00374709_m1 |
| Human SOX6 | Hs00264525_m1 |
| Human S100β | Hs00902901_m1 |
| Human AGGRECAN (ACAN) | Hs00153936_m1 |
| Human COL2A1 | Hs00264051_m1 |
| Human COL9A1 | Hs00932129_m1 |
| Human COL11A1 | Hs01097664_m1 |
| Human RUNX2 | Hs00231692_m1 |
| Human MMP13 | Hs00233992_m1 |
| Human GAPDH | Hs02758991_g1 |

**Sup.Table.1.** List of qRT-PCR Taqman® probes used in this study.

**Lin.Sup.Table.2.**

|  | | **Cytodex 1** | | **Cytodex 3** | | **SphereCol** | | **Cultispher-S** | |
| --- | --- | --- | --- | --- | --- | --- | --- | --- | --- |
|  |  | Day 0 | Day 28 | Day 0 | Day 28 | Day 0 | Day 28 | Day 0 | Day 28 |
| Chondrogenic markers | Sox9 | 1.20 | 2.23 | 1.28 | 2.22 | 1.28 | 0.68 | 0.97 | 2.47 |
|  | Sox5 | 1.52 | 1.44 | 1.22 | 1.17 | 1.22 | 0.87 | 1.20 | 1.72 |
|  | Sox6 | 1.13 | 2.61 | 1.05 | 2.41 | 1.05 | 1.37 | 1.00 | 2.38 |
|  | S100β | 0.98 | 2.05 | 1.04 | 2.77 | 1.04 | 2.05 | 0.88 | 1.77 |
|  | ACAN | 1.19 | 2.13 | 0.94 | 2.09 | 0.94 | 0.68 | 0.61 | 3.49 |
|  | Col2A1 | 1.16 | 11.3 | 9.46 | 9.41 | 9.46 | 1.47 | 2.68 | 16.2 |
|  | Col9A1 | 1.11 | 11.3 | 1.17 | 8.94 | 1.17 | 2.00 | 0.85 | 14.7 |
|  | Col11A1 | 0.99 | 3.11 | 0.99 | 2.86 | 0.94 | 1.11 | 1.11 | 3.70 |
| Hypertrophic markers | Runx2 | 1.13 | 1.56 | 0.91 | 1.04 | 0.91 | -3.39 | 0.80 | 2.46 |
|  | MMP13 | 1.07 | -13.4 | 1.01 | -24.0 | 1.01 | -13.7 | 0.73 | -3.35 |
|  | Sox9/Runx2 | 1.06 | 1.43 | 1.41 | 2.13 | 1.41 | 2.31 | 1.21 | 1.00 |

**Sup.Table.2.** Gene expression profiles of critically-defined heMSC-microcarrier constructs across different microcarrier types at day 0 and 28 of differentiation. All numbers shown indicate the fold changes of heMSC-microcarrier constructs over that of cells-only counterparts at indicated timepoints.
